# Newly evidence across the world on how climate financing helps in ensuring a greener future

**DOI:** 10.1016/j.heliyon.2024.e34779

**Published:** 2024-07-17

**Authors:** Zehao Liu, Chi Paan

**Affiliations:** a Agricultural Economics, Texas A&M University, College Station, TX, 77845, USA; b Wuhan University, China

**Keywords:** Climate financing, Green future, Climate

## Abstract

Investing in climate change mitigation and adaptation has become a promising approach to address the negative impacts of climate change on the environment and to encourage efforts to adjust to changing conditions. Climate funding is a kind of foreign aid that seeks to reduce carbon dioxide emissions and promote ecologically sustainable economic development. However, it is not quite clear to what extent it helps recipient countries transition to low-carbon pathways. This research examines the worldwide flow of climate finance from 1999 to 2017 to determine the impact of climate funds on reducing carbon emissions and promoting sustainable development in recipient countries. The data suggest a potential correlation between climate funding and the reduction of carbon emissions, with investments in mitigation seeming to have a greater impact than spending on adaptation. Furthermore, countries that have achieved significant economic progress and Small Island developing states stand to benefit more from climate aid aimed at reducing emissions. This is due to the fact that both sorts of nations exhibit greater rates of economic growth. These results are significant for policymakers, who may use them to accelerate the transition to net-zero combustion levels and enhance environmental sustainability.

## 1 Introduction

Due to the buildup of atmospheric carbon dioxide, environmental degradation, and the greenhouse effect have occurred, posing formidable obstacles to resolving climate change and its accompanying problems. As defined by the United States Environmental Protection Agency, environmental sustainability has swiftly become a focal point for international organizations and national governments, making environmental sustainability research a priority. The worldwide community has taken several steps to enhance people's lives today and in the future by reducing the effects of climate change. In recent years, we have seen extraordinary speeds and magnitudes of change throughout the climate system; between 1905 and 2019, the Epidemiology of Disasters (Database recorded a total of 13,589 climate-related catastrophes. Most of these disasters were in the form of storms, thunderstorms, drought, landslides, earthquakes, and severe weather. These disasters were directly responsible for the deaths of 20 million people and the loss of USD 4130 trillion in GDP in the affected countries. Prevention of environmental deterioration is more critical than ever in light of the increasing threats posed by climate change.

The main theme of the article is to analyse the influence of climate finance on the reduction of carbon emissions and the promotion of sustainable development in countries receiving funding. This analysis examines the global movement of climate financing between 1999 and 2017, emphasizing a possible connection between funding for climate initiatives and the decrease of greenhouse gas emissions. The research highlights that investing in mitigation measures has a more substantial effect compared to spending on adaptation. It also reveals that economically advanced countries and Small Island Developing States have large advantages from these expenditures. The study seeks to examine the impact of climate funding on promoting ecological responsibility and lowering CO2 emissions, particularly in vulnerable developing nations, via the formulation of hypotheses. In summary, the report highlights the crucial importance of climate financing in speeding up the shift towards low-carbon paths and improving environmental sustainability on a worldwide scale.

However, according to Ref. [1], investment from wealthy nations still needed to reach US $ 100 billion yearly by that Year. The fundamental cause was the negotiators' inability to agree on a standard metric to evaluate the commitment's efficacy [2]. [3] argued that the agreement's effectiveness could only be determined with information on how much money would be provided to each plan, such as loans and grants. Because the deal does not specify how much money will be put into each program, this is the case. A new target for climate finance by 2025 with an annual value of at least USD 100 billion is required under the Paris Agreement, as stated by Ref. [4]. Research commissioned by the UN Secretary-General found that donors have exaggerated their contributions to climate change mitigation by $3 billion to USD 4 billion. The United Countries Framework Convention on Climate Change has forced industrialized nations to classify all available programs as “climate finance” despite no agreed-upon definition, method, or guidelines for choosing such funding [5].

There has been a substantial amount of practice and cooperation on a global scale in reducing carbon dioxide emissions to realize the goal of achieving ecological responsibility. This decrease is accomplished via a wide variety of various methods. Additionally, “international low-carbon technology” refers to the many ways CO2 emissions are reduced. These innovations, which contain hydroelectric generation, photovoltaics, solar and wind, and sustainable sources, have as their central focus the improvement of energy efficiency and the use of alternative energy sources that are less polluting [6]. Finally, the Nationally Adjusted Commitment is the new standard for determining whether a country conforms to the Paris Agreement. This strategy relies on National Determined Contributions expressed by protocol states to reduce the number of greenhouse gases released into the atmosphere globally [7].

Despite widespread attention paid to tried-and-true methods for addressing climate-related issues with environmental quality, the academic community has largely overlooked a rapidly growing field: climate finance. Since industrialized countries have provided financial help to developing countries as part of their efforts to counteract and adapt to the consequences of climate change [8]. Financing for mitigation and adaptation has been reinforced as a significant priority in international climate talks since it is one of the most effective ways to help low-income nations adapt to climate change [9]. Investments in low-carbon and climate-resilient development are what we mean when we talk about “climate financing” in this context. The positive impacts of these cash flow on reducing and adapting greenhouse gas emissions help to achieve minimum carbon limits to promote ecologically sustainable growth [10,11]. Climate funding was developed as a new incentive for low-carbon growth and a mechanism to aid in reducing global emissions to assist developing countries in balancing the need to maintain economic development with the need to reduce greenhouse gas emissions. Because developing countries tend to lack the means to address issues of environmental sustainability, this was intended to be an essential first step.

To reduce emissions and protect the Environment, donor countries transfer money to developing countries via a mechanism known as “climate finance” [12]. Two main categories of climate finance may be defined by the results they are meant to achieve. Adaptation funding assists in preparing for and responding to the effects of climate change, whereas mitigation finance works to curb the production of greenhouse gases via measures like increasing investment in renewable energy and decreasing deforestation [13]. The world's poorest nations might benefit significantly from a new approach to mitigating climate change and preventing future environmental degradation: climate financing, especially investment for mitigation. As you can see, the evaluation's setting is of paramount importance.

The study of climate finance from a variety of perspectives has recently increased. Many researchers have looked at that impact. Like the Environment's impacts on adaptation capacity regarding recipient nations and the effectiveness of the world's reaction to the Environment [14,15]. Above all of our intentions, there needs to be more data on the impact of environmental investment on the outcomes of receiving nations regarding CO2 outlets; the link among environmental investment and fewer excretions needs to be clarified.

The decrease in carbon dioxide emissions rate measures climate money's success in enhancing environmental quality in developing nations. We examine the assumption that small island developing states that get external funding, such as climate money, would improve their performance in preserving the Environment and engaging in low-carbon practices. In addition, recipient nations' degree of economic and monetary development affects the correlation between CO2 emissions and climate financing. For this reason, we look at the economic and financial growth stage that heralds an era of improved efficacy for climate finance in terms of lowering emissions. Based on our empirical research, we know that giving money to developing countries to help them adapt to climate change has a minor impact on emissions than giving money to help them reduce their emissions, but both have a positive effect. The planned heterogeneity study may help Small Island developing states better allocate climate funds to reduce CO2 outlets and move toward a less one. As a result, countries experiencing both fast economic development and limited access to traditional financial resources stand to benefit the most from climate financing's influence on CO2 emissions. This is because the financial industry in these nations has more room to grow.

The four main contributions from the analysis are all significant advancements in the field. To begin, we expand on recent studies on the growing importance of climate finance by looking into how weather fund affects the ecological responsibility of developing nations, where the vast majority of previous research on funding has focused on the efficiency and distribution of climate money [16]. However, there needs to be more empirical research into this factor in the scholarly literature. With us, findings suggest that rethinking assistance mechanisms like public funding in light of the connection between air sustainability and environmental issues can help developing countries make more substantial progress toward sustainable development. The second way we improve transparency is by classifying climate funding according to their goals. Three types of funds fit this description: public climate financing, mitigation funds, and adaptation funds. These indicators provide data for a more thorough evaluation and understanding of the subject. Third, given the disparity in environmental vulnerability between SIDS and non-SID countries, the sample countries are analyzed to see whether there is a unique connection between the two sets of nations. This is done because of each stage of nations' distinct climate dangers. Our research offers novel insight into the environmental sustainability of these developing countries. Finally, to thoroughly understand the underlying channel, we include economic growth and financial development contributions.

The following paragraphs provide an overview of the organization of the remaining parts of this paper. In the second section, we examine the relevant literature. The third section will focus on how data was collected and analyzed for this research. The findings are summarized in Section 4. The results are presented in Section 5, followed by a discussion of their policy implications.

## 2 Literature review

### 2.1 Ecological stability

An essential component of environmental sustainability is climate action, which aims to reduce the effects of global warming caused by emissions of greenhouse gases. Ecological sustainability is a broad concept encompassing various environmentally responsible practices. In particular, for emerging nations, which usually have a shortage of funds and equipment to deal with environmental devaluation, addressing climate change presents an ideal way to enhance the ecological condition and less-carbon advancements strategies simultaneously. This is prominently true because climatic changes are expected to worsen shortly [17].

In the body of research that has been done on the topic, a variety of tried-and-true approaches to improving environmental sustainability have been recorded. To get things started, the carbon trading market is one of the strategies with the broadest support base for enhancing emerging countries' environmental quality. This covers selling rights to emit greenhouse gases and other financial operations and transactions [3]. On the other hand, many studies have proven that trading carbon fails to achieve its objective and creates operational challenges [18]. To achieve the goals of equality in ecological maintenance and to close the technological difference in emerging nations, contribute to an overall improvement in the quality of the Environment all over the world. In particular, countries can participate in programs that aim to lower greenhouse gas emissions in developing nations by selling and purchasing credits via the Clean Development Mechanism [19]. The technique of technological exchange among states has been slowed by disagreements between advanced and emerging nations over their stand on technical discussion, as well as the absence of critical linkage of CTM on developed nations. However, it allows backing for transferring ecological variations to emerging nations [20,21].

Alternative means of monetary cooperation in international environmental agreements may be accomplished via climate funding. Most statistical economic surveys highlight how climate finance affects recipient countries’ ability, and financial goals [8], like the factors that influence ecological funds reservation [22,23]. According to the Climate Policy Initiative, “climate finance is a difficult program of national decision-making for both donor and recipient countries,” and this is because the process includes a wide variety of government departments [24,25]. Climate-related financial flows could be wasted due to projects and programs that are inadequately conceived or carried out [26]. As a consequence of this, it is difficult to predict how climate financing will influence the health of the Environment.

### 2.2 The process of formulating suppositions

Climate finance is a crucial component of international financial instruments that plays a significant role in defining the achievable degree of environmental sustainability. As to the study conducted by Ref. [15], climate funding has started a new stage by providing help to poor countries in their efforts to combat the impacts of climate change and achieve sustainable development. This was achieved by offering monetary aid to these nations. More precisely, funds allocated for mitigation may support poor countries in transitioning to social systems that generate lower levels of carbon emissions [18]. [8,27] contend that a substantial augmentation in financial resources allocated to climate change mitigation and adaptation is important for the establishment of an ecologically sustainable society in the current period. This might be achieved by restructuring the energy framework [9,28]. examine the allocation of climate money for the purpose of funding renewable energy production and biosphere preservation. They use a propensity score matching approach to analyses these flows. The researchers determined that the nations that received “fast-start” climate money in 2010 effectively reduced their emissions of greenhouse gases and augmented their utilization of renewable energy sources. Here is the provisional hypothesis that we have formulated based on these discussions.

#### 2.2.1 First supposition

Climate financing, which will help ensure the long-term viability of the planet's biota, would be an unacceptable effect on CO2 outlets produced by emerging nations.

The demands of the small island developing nations are among the most pressing of all the growing countries. As a result of their economies being so dependent on just one sector, many small islands developing states are particularly susceptible to environmental deterioration and severe losses [29]. The administrations of small islands are primarily dependent on foreign help and collaboration due to their extreme exposure to natural catastrophes and isolation from the rest of the world [30]. The financial issue makes it challenging for Small Island Developing States to achieve sustainable development and create resilience [23]. Concluding their study of the Solomon Islands, communities may reap benefits from implementing climate change adaptation strategies that are also intended to aid communities in moving through different phases of development. After using these procedures, we got these outcomes [31]. Examines the international aid received by small island developing nations for renewable energy from 1971 to 2014, shedding light on the role of foreign support in implementing renewable energy in these countries. Comparable work is done by Ref. [19] concerning the function of foreign aid in the energy industry. Through climate funding, the international community may assist small island developing states in overcoming these obstacles. “Climate money” is a relatively new kind of international aid for environmental sustainability. Therefore, this Supposition must be considered.

#### 2.2.2 Second supposition

Compared to other developing nations, the small island developing states are the ones in whom financial assistance for climate change has a much more significant impact on reducing emissions.

Many experts in academia have weighed in on ecological funds and CO2 outlets' effect on economic and financial development. For developing countries to be eligible for money related to climate change, they must first overcome various obstacles. Problems emerge due to factors such as inadequate flexibility at the technological level, inadequate access to information and markets, and institutional shortcomings. These factors make it more difficult for less developed countries to acquire cutting-edge technologies [26]. It has been suggested [5] that developing countries benefit from international financial cooperation if they are better equipped to deal with the trade-offs in striking a healthy balance between economic development and environmental conservation [25]. Their findings concluded that developing countries have a lower capacity to deal with trade-offs than developed countries. Because of this, the third hypothesis is the logical next step for us to take.

#### 2.2.3 Third supposition

Economic growth and financial sector development will likely influence the efficiency of climate financing in reducing carbon dioxide emissions.

## 3 Research methodology

### 3.1 Design of the proposed system and estimates

To construct an accurate model that can determine the effects of climatic funds on ecological conditions, firstly, essential for a solid understanding of the components that might impact ecological conditions. Environmental influence is broken down into population, prosperity, and technological advancement. Specific practical theory may be expressed in the following manner, which is given below,

(1) $$CO2_{it}=\beta_{0}+\beta_{1}{CF}_{it}+\gamma {Controls}_{it}+\delta_{i}+\phi_{t}+\varepsilon_{it}\;\ldots$$

In this case ,i=1,…,N indicates the nations receiving aid, and t=1,…,T is the timeframe in question. CO2it refers to total annualized global Co2 emissions as a dependent variable. To measure finance, we use the proportion of total climatic investments, emissions reduction funding, and climate change mitigation and adaptation funds to the gross domestic product of the nation receiving the funds (CFit,theprimarypredictiveparameter). Population (POP), value added (IVA),FDI, and energy intensity are all examples of control factors that may be used to modify a country's overall carbon emissions profile (EI).

Finally, δi and ϕt set the basis of nation and period, respectively; εit represents the error term.

### 3.2 Justification of data

This study analyses the effects of environmental funds on ecological conditions in receiving nations by compiling a large group of 129 developing countries from 1999 to 2017. We can quickly achieve the Kyoto Protocol's target of a worldwide peak in greenhouse gas emissions by 2030 [22]. It was the Kyoto Protocol that gained attention for its pioneering commitments to reduce emissions on a worldwide scale. We selected an extended period for which data is now available to assess climate money's effectiveness in enhancing environmental quality via emission reductions.

There are three key reasons why we choose to focus on poorer countries. To start, the most significant obstacles to global fairness in climate policy and environmental preservation come from developing nations, which are only beginning industrialization. Another factor is that underdeveloped countries do not have what it takes to repair ecological damage and mitigate the consequences of climate change. Third, most climate aid is given to developing countries because of economic inequality. These countries must balance encouraging industrialization with safeguarding their natural resources.

Carbon dioxide emissions from human activities significantly contribute to atmospheric concentrations of these gases. This is because carbon dioxide emissions are a proxy for the ecological damage wrought by industrialization. One of the leading causes of environmental degradation due to climate change has long been represented by Refs. [8,14]. According to the findings of (Y. Zhang, Zhang et al., 2021), rising global temperatures and deteriorating weather patterns are mostly linked to human-caused carbon dioxide emissions. To that aim, we use carbon dioxide emissions from WDI as a primary dependent variable in environmental sustainability assessments.

Comparing the global climate fund, climate mitigation fund, and climate adaptation fund to GDP provides a rough estimate of climate finance. Greenhouse gas concentrations in the atmosphere must be maintained below safe thresholds to avoid any unfavorable impacts on the climate system from human activities. Most of the money allocated to projects to reduce greenhouse gas emissions goes toward low-carbon transportation systems and renewable energy sources like solar photovoltaics and onshore wind farms.

Due to the lengthy time it takes recipient countries to complete projects funded by this kind of funding, a two-period technique is employed to limit the irregular influence of climate money. Generally speaking, there are three stages to every planning endeavor: A country that receives climate finance in year t would utilize the differences-in-differences method to divide the money between that Year and the next. Funds received in Year t and Year t minus one may be included in the total; climate finance that is not received in a given year is ignored. The same logic may be applied to the many ways we may alter the passage of time.

We also factored in national-level characteristics such as the destination country's population size, industrial value, foreign investment levels, and energy intensity of its economy. Rapid population expansion is detrimental to natural systems. Others, however, maintain that a more significant population is beneficial for the Environment since it hastens urbanization, which increases the effectiveness of public amenities and fosters industrial agglomeration, thereby decreasing fuel intake and decreasing outlets [32].

Considering value added in the industry helps mitigate the effects of economic growth and industrialization, both of which are associated with a rise in carbon emissions. Foreign direct investment will likely capture potential technological developments, especially as emerging nations cannot bear expensive steps in combating the ecosystem. Finally, because high-energy use to accept climate money for renewable energy projects, EI may be difficult for countries that rely significantly on fossil fuels for economic prosperity [12].

Table 1 details the many sources of the data collected for this analysis. Table 1 summarizes the 129 emerging countries included in this study, classifying them into the 50 SIDS and 83 other countries. The United Nations has recognized the Small Island Developing States since 1992, when 58 were determined based on geographical, socioeconomic, and climatic criteria [24]. Small island developing states may benefit significantly from climate finance, which makes accessible a significant amount of sanctioned funds to aid in resilient growth to climate change's consequences. Examples include funding for measures to adapt to climate change or reduce carbon emissions. The small island developing states are an excellent area to evaluate the effectiveness of current climate finance programs because of the potential impact climate finance has had there.

**Table 1:** Descriptive statistics.

Variables	Obs-	Mean	Maximum	Minimum	Standard Deviation
**Panel I Samples**					
Carbon dioxide	1580	1.912	15.721	0.031	2.312
CF	1580	0.003	0.054	0	0.002
AF	1580	0.002	0.059	0.1	0.008
IVA	1580	0.002	11.68	3.94	1.724
HI	1580	6.851	36.98	2.144	5.918
SID	1580	0.187	1.2	0.2	0.41
MF	1580	0.02	0.049	0.3	0.006
POP	1580	15.05	22.04	10.49	2.902
FDI	1580	3.463	96.98	−9.28	7.286
FD	1580	0.422	2.602	0.4	0.39
**Panel II: SIDS**					
CF	280	0.048	0.075	0.3	0.02
CO2	280	1.867	5.681	0.133	1.223
POP	280	12.367	17.186	12.493	2.43
AF	280	0.068	0.065	0.2	0.008
FDI	280	6.038	43.093	−9.275	0.872
IVA	280	8.98	11.8	7.286	0.678
FD	280	0.46	0.845	0.001	0.336
HI	280	6.287	15.54	3.056	3.75
MF	279	0.008	0.063	0.1	0.006
**Panel III: Non-SIDS**					
CF	1197	2.905	0.004	0.3	0.0354
CO2	1197	1.804	3.557	0.031	16.464
POP	1197	17.786	2.334	12.13	22.045
AF	1197	0.3	0.002	0.1	0.002
FDI	1197	5.178	5.832	−4.308	84.898
IVA	1197	8.901	2.213	5.98	13.678
FD	1197	0.131	0.304	0.032	2.603
HI	1197	8.123	6.198	2.143	35.787
MF	1097	0.002	0.1	0.2	0.033

Our sample data suggests that the CO2 emissions range from 16.685 to 0.016. The data collected led to this conclusion. There is a statistically significant difference between SIDS and non-SIDs in this way of CO2 emission in the subsample groups, indicating that non-SIDs bear a disproportionate burden in the effort to curb pollution contributing to global warming. This is because non-SIDS contribute more to global emissions than SIDS does. According to the ratio of climate money to GDP, small island developing states depend on climate funding more than other countries. Most of the money spent on adaptation was by minor island-creating conditions, which had a 63.5 % ratio to GDP. Since adjusting to climate change is prioritized, this is the case. Our investigation has uncovered three main reasons for this behavior. Before further action is taken, it is necessary to acknowledge that the costs of climate adaptation in small island developing states are high compared to other countries gross domestic product. Second, even if small island developing states contribute a small amount to global carbon dioxide emissions, affluent nations should support climate action [3]. Finally, tiny island developing states have struggled for a long time due to a lack of accessible financial resources, which has been the biggest obstacle to sustainable progress. To lessen the negative consequences of climate change, the international community can financially assist the tiny island developing states.

## 4 Discussion and results

### 4.1 Recognizing the significance of findings

“Climate finance” means sending funds to developing countries to aid in their fight against climate change and to boost green economic growth. Most developing nations are still in the first phases of industrialization. Therefore, carbon emissions are often integrated into their manufacturing procedures. Our research aims to determine whether and to what extent climate money improves environmental quality in developing countries. Furthermore, we want to conclude the potential distribution and use of climate money in the future to enhance environmental quality.

Table 2 displays the estimated outcomes of the model, including the panel fixed effect (1). The critical climate finance variables have implications like the disaggregated estimates shown in columns (1–3). Based on the data in the first column, providing financial support to combat climate change would result in a significant cutback in the quantity of carbon dioxide emissions generated by the poor receiving nations. This supports the research of [33] that climatic financing may stimulate power production, lessen reliance on fossil fuels, and contribute to lower greenhouse gas emissions. Thus, our research shows that climate finance is a powerful tool in the battle against environmental problems and global warming.

**Table 2:** Climate finance effects on CO2 emissions.

	1	2	3
CF	−10.775		
	6.112		
MF		−15.678	
		7.987	
AF			−12.654
			6.967
POP	−1.578	−1.456	−1.546
	0.699	0.698	0.678
IVA	0.407	0.4	0.472
	0.2	0.21	0.23
FDI	0.599	0.599	0.599
	0.007	0.007	0.007
HI	0.6	0.61	0.6
	0.022	0.022	0.022
Obs−	1568	1568	1568
R−2	0.935	0.935	0.935

Implementing climate finance, which reduces environmental risks, might lead to a more significant infusion of cash into environmentally responsible firms. Without this help, developing nations would have to weigh the benefits and costs of promoting economic growth while protecting the Environment [34]. According to this theory, low-income nations are encouraged to experiment with low-carbon strategies for economic development by receiving climate subsidies. Funding support is crucial for lowering carbon emissions, bettering the Environment, and realizing economies of scale, all of which are good for the economy [1]. The “spillover” effect resulting from technological progress may be the root of the positive correlation. Climate finance's emphasis on low-carbon technologies has boosted renewable energy sources, improving environmental results [35].

More significant results are attained if all climate-related monies are divided into adaptation finance and mitigation funding. These buckets represent the wide variety of people who may be given grants. Spending money on adaptation to climate change has a higher rate of return than spending money on reducing the impacts of climate change. Money set aside to deal with the repercussions of climate change is called “adaptation finance,” while money set aside to reduce those effects is called “mitigation finance.” According to Ref. [2], With more money going toward reducing greenhouse gas emissions, decarburization in high-emissions industries will likely progress more quickly.

The bulk of the estimates for these three independent variables will be very significant from the preliminary data. Since fewer carbon emissions are produced due to growing populations, the POP coefficient is positive. The introduction of cutting-edge transportation methods contributed to this undesirable outcome. Using more and more fossil fuels has resulted in a significant rise in emissions, which is directly attributable to the increasing pace of industrialization. One potential explanation for this trend is that countries with higher levels of automation are less likely to pass regulations promoting renewable energy sources.

Foreign direct investment will likely reduce greenhouse gas emissions. These findings align with what we know about foreign direct investment and environmental quality. The economic theory has a rich history that labels developing countries as “pollution havens.” An argument states that these countries may profit economically from the international investment while implementing stricter environmental rules. Since high energy intensities encourage using conventional fossil fuels, which raises carbon dioxide emissions, they are positively connected. Since humans rely on nonrenewable energy sources, their energy consumption and intensity significantly contribute to greenhouse gas emissions. Recent studies imply that increased energy intensities contribute to rising carbon emissions in developing countries [17].

### 4.2 Robustness check

Our empirical data were rigorously tested to confirm their validity and reliability using several additional procedures. We should have included information from the five newest industrialized nations (Brazil,Russia,India,China,andSouthAfrica) to lower the chance of spotting any anomalies (BRICS). Carbon dioxide emissions are high because of the BRICS nations' rapid economic expansion, expanding populations, and generally stable political systems. This means that BRICS nations use more energy than others in the world. It is impossible to overestimate the significance of the *BRICS* countries to global economic growth or the difficulty of striking a balance between ecological safety and economic advancement in their nations [36]. It is necessary to consider that various nations' development depends on diverse production systems when estimating the consequences of environmental degradation. The inclusion of *BRICS* in the estimates that favor the beneficial influence of ecosystem investment on ecological conditions may account for any discrepancies between the main results in Table 2, Table 3.

**Table 3:** Climate finances effects on CO2 emissions without bricks

	1	2	3
CF	−9.444		
	4.132		
MF		−13.243	
		7.211	
AF			−13.856
			16.545
POP	−1.122	−1.155	−1.344
	−0.633	−0.6321	−0.664
IVA	0.278	0.277	0.245
	1.63	1.64	1.62
FDI	0.005	0.005	0.005
	0.007	0.007	0.007
HI	0.55	0.56	0.55
	0.021	0.024	0.022
OBS	1502	1502	1502
R2	0.922	0.922	0.922

Second, as was said before, it takes time for governments to complete the programs that use money designated for climate change. Concerns about the dynamic impact shown in the prior study were alleviated by a supplementary test that we carried out using a four-period smoothing method. The following information, which is shown in Table 4, agrees with our past results.

**Table 4:** RobustnessCheck;AlternativesmoothingStrategy.

	1	2	3
CF	−12.1		
	5.554		
MF		−21.342	
		9.443	
AF			−14.232
			8.142
POP	−1.652	−1.673	−1.65
	0.745	0.74	0.741
IVA	0.401	0.421	0.403
	0.213	0.213	0.213
FDI	0.005	0.005	0.005
	0.007	0.007	0.007
HI	0.064	0.063	0.064
	0.022	0.022	0.022
Obs	15.63	15.63	15.63
R−2	0.944	0.944	0.944

Third, our first data suggests there may be a negative correlation between climatic financing, Gross Domestic Product, and CO2 emission, which is cause for concern. This would lead to more money being given to countries with high emissions so they can help decarbonize their economies. We run a basic indigeneity test using a period behind independent variables to ease some of the worries raised by the indigeneity issue. Table 5 displays the results from the exact estimate, which corroborate our previous findings and strengthen the validity of the conclusion reached in this study.

**Table 5:** RobustnessCheck:EndogeneityconcernswithOneYearlaggedIndependent.

	1	2	3
L1.CF	−6.884		
	2.332		
L1.MF		−12.845	
		5.454	
L1.AF			−8.876
			4.115
Controls	yes	yes	yes
Obs	1422	1422	1422
R2	0.899	0.899	0.899

Finally, a new analysis is presented using the GMM framework that deals with the Endogeneity and continuity impacts of time in a two-stage procedure. The outcomes are shown in Table 6. Carbon emissions were shown to be reduced with climate finance aid, with mitigation money more successful than adaptation financing. The findings of the robustness tests confirmed our previous assumptions.

**Table 6:** Robustness Check; Endogeneity Concerns two−step system GMM.

	1	2	3
L1.CO2	0.9123	0.921	0.942
	0.004	0.003	0.002
CF	−7.543		
	0.689		
MF		−18.767	
		1.07	
AF			−12.567
			3.087
Controls	yes	yes	yes
Obs	1398	1398	1398
AR2−P−value	0.772	0.775	0.773
Sargan−Pvalue	0.111	0.13	0.021

### 4.3 Dissimilarity in outcomes

Small island developing states are unique among the world's countries when dealing with global environmental issues like ecosystem variations. SIDS are especially susceptible to ecosystem variations and ecological devaluation because of their proximity to the shoreline. For many nations, the probability of being submerged by rising sea levels due to increased global temperatures caused by humans poses an imminent danger to their existence. The particular socioeconomic aspects of small island developing states make adaptation to climate change and recovery from the effects of natural disasters tenfold more difficult for *SIDS* than for other countries. Small island developing states have fewer resources to invest in adaptation measures and climate change mitigation due to their smaller economies and higher dependence on imported commodities. A problem arises because tiny island developing nations are particularly vulnerable to the adverse effects of climate change [10]. Small island developing states and other developing countries emit much more significant and significantly different amounts of carbon dioxide. We use this information to strengthen our case for more funding related to climate change.

Data in Table 7 suggests that with access to climate funds, small island developing states may be able to cut their carbon dioxide emissions more effectively than other developing countries. Our findings support the second hypothesis, suggesting that the more a country relies on foreign aid, its vulnerability to external threats increases. They mainly depend on outside assistance because of their isolated positions, which increases their susceptibility to natural disasters [20]. Despite climate finance's advocacy of universal support for assistance funding, SIDS gets a disproportionately small amount of the total funds allocated to these purposes. When looking at climatic financing flows as a percentage of Gross Domestic Product/capita, as shown in Table 1, tiny island developing countries benefit the most monetarily. Small island developing nations have been able to make their agricultural practices, biodiversity, and infrastructure more robust to the effects of climate change thanks primarily to assistance from this climate effort. This helps them to cut down on fossil fuels and boost their use of renewable energy.

**Table 7:** Heterogeneity analysis and Non-SIDS.

	1	2	3
Panel A:			
CF of Non-SIDS	−14.55		
	16.88		
CF of SIDS	−9.98		
	3.98		
Panel B:			
MF of Non SIDS		−15.34	
		17.77	
MF of SIDS		−15.001	
		5.223	
Panel C:			
AF of Non SIDS			−26.878
			33.989
AF of SIDS			−11.675
			5.887
Controls	yes	yes	yes
OBS	1499	1499	1499
R2	0.877	0.877	0.877

### 4.4 Improved economic and monetary forecasts

To further understand the impact of economic growth, we segmented our samples into nations with less developed economies, those in the medium-income range, and those in the upper middle-income field. Table 8 presents the findings of this study. The results show that economically developed nations are more likely to successfully employ climate change funding to reduce carbon emissions and save the Environment. These findings provide supporting evidence for the third hypothesis, which postulates the existence of a conditional impact in economics. To maintain their populations' high living standards and raise their electricity, emerging nations with a higher per capita income may look into alternative energy sources.

**Table 8:** HeterogeneityAnalysis:EconomicDevelopment.

	1	2	3
PanelA:			
CF/GDPofLow−IncomeCountries	−10.232		
	13.443		
CF/GDPofLowermiddleCountries	−25.776		
	17.897		
CF/GDPofUppermiddleCountries	−12.767		
	4.989		
PanelB:			
MF/GDPofLow−IncomeCountries		−14.554	
		15.998	
MF/GDPofLowermiddleCountries		−40667	
		23.455	
MF/GDPofUppermiddleCountries		−15.233	
		6.776	
PanelC:			
AF/GDPofLow−IncomeCountries			−30.221
			35.221
AF/GDPofLow−IncomeCountries			−25.998
			18.111
AF/GDPofLow−IncomeCountries			−17.223
			7.554
Controls	yes	yes	yes
Obs	1511	1511	1511
R2	0.821	0.821	0.821

Furthermore, much of the money spent to combat climate change is helping middle-income countries reduce their emissions. At the same time, low-income nations receive very little return on their investment. Investment in tackling climate change helps the economy grow, which is good for the planet. This result is in line with earlier studies [21] that found that economic growth and technical progress contribute to carbon emission at different societal levels of affluence. The results of this study agree with those of its predecessors.

## 5 Conclusion and policy implications

According to experts in international climate policy, economic development cannot be sustained if the environmental quality is reduced. This school of thinking has advanced the cause of global climate justice for emerging nations by facilitating mechanisms such as climate finance. It is still being determined how much of an effect climate finance will have on environmental quality concerning carbon dioxide emissions, even though it is a novel ecological investment. The impact of ecosystem funding on CO2 emissions in 129 developing nations from 1999 to 2017 is analyzed. We also look at how climate funding affects small island developing states differently than other countries. The conditional impact of climate finance elucidates the connection between foreign assistance like climate funding and environmental quality by considering economic and financial growth.

Our first empirical findings indicate that climate aid, especially mitigation assistance, may help lower carbon dioxide emissions in emerging nations. Second, small island developing states have a more significant opportunity to reap the rewards of climate funding to curb emissions than other developing nations. Finally, the effects of climate finance on economies and financial systems at various stages of development are unique. If the receiving country accelerates its economic growth, the climate money it receives to enhance environmental quality via low-carbon policies will significantly impact it. When it comes to economic growth, however, climate investment is more likely to improve ecological quality in less developed nations.

### 5.1 Policy implications

The results of our analysis have diverse and substantial policy ramifications for global ecosystems in the long run. Research highly advise rich nations to prioritize their dedication to actively encouraging compliance with climate finance since it can greatly influence carbon emissions in recipient countries. In order to strengthen funding for environmental initiatives, it will be imperative to get contributions from various entities, such as the federal government, private sector, corporations, and municipalities. The results of our research suggest that Small Island Developing States (SIDS) have a higher level of efficiency in utilising climate money compared to other developing countries. This highlights the need to allocate additional resources to guarantee the effective reduction of emissions.

It is essential to acknowledge that recipient nations must possess significant economic growth in order for climate money to effectively accomplish its intended goal of carbon reduction. Authorities should increase the allocation of resources towards bolstering economic growth to enhance their ability to absorb climate money. Considering their outsized role in causing global warming, it is essential to prioritize poor nations when allocating funds for climate-related initiatives. Policymakers must recognise the importance of ecosystem variety in global efforts to achieve carbon neutrality objectives. Every policy should prioritize the execution of climate funds, which may be used to reduce the impact of climate change and enhance environmental conditions. It is crucial to analyse the means by which poor nations might get climate finance and formulate appropriate climate policies to tackle the difficulties posed by climate change efficiently.

### 5.2 Future research avenues

Finally, our inquiry sheds light on the impacts of climate finance on environmental sustainability in emerging nations and suggests future research avenues. To begin, energy exporters and energy importers may pay different prices throughout the low-carbon transition. Classifying developing countries by their principal energy source may help with climate budget allocation. Second, the word “environmental sustainability” covers various ideas. This category falls under human well-being, environmental sustainability, and responsible use of limited resources. Another productive topic for future study is the effects of climate finance on many aspects of ecological sustainability.

## Ethics approval and consent to participate

Not applicable.

## Consent for publication

All of the authors consented to publish this manuscript.

## Funding

Funding Information is not available.

## Data availability

We collected relevant data from World Bank open data available at https://data.worldbank.org/. For any further query on data, corresponding author at email address naomiliu@tamu.edu may be approached.

## CRediT authorship contribution statement

**Zehao Liu:** Writing – review & editing, Writing – original draft, Visualization, Project administration, Methodology, Data curation, Conceptualization. **Chi Paan:** Writing – review & editing, Writing – original draft.

## Declaration of competing interest

The authors declare that they have no known competing financial interests or personal relationships that could have appeared to influence the work reported in this paper.
